# Extending social presence theory: social presence divergence and interaction integration in online distance learning

**DOI:** 10.1007/s12528-022-09325-2

**Published:** 2022-06-16

**Authors:** Joshua Weidlich, Derya Orhan Göksün, Karel Kreijns

**Affiliations:** 1 DIPF Lebniz Institute for Research and Information in Education, Frankfurt, Germany; 2 Adiyaman University, Adiyaman, Turkey; 3 Open University, Heerlen, The Netherlands

**Keywords:** Social presence, Distance education, Online learning, Social presence theory, Divergence, Interaction integration

## Abstract

Social presence is an important concept for understanding psychosocial processes in learning scenarios that make extensive use of mediated communication like online distance learning. Despite this centrality, a coherent and nuanced theory of social presence is yet to emerge from the literature. Past research has shown associations with desirable affective variables like satisfaction and perceived learning, yet our knowledge as to when and for whom these effects are expected is still very limited. By introducing two contextual explanatory variables, we provide the means toward a more mature theory of social presence. The first variable, social presence divergence, relates students experiences to their preferences, yielding three distinct scenarios: too little, too much, and just the right amount of social presence. The second variable, interaction integration, considers the centrality of social interaction in the learning scenario, suggesting that this functions as a moderator. In a sample of teacher education students (N = 305), we find evidence that these variables interact with social presence and affective dependent variables as expected. These results add nuance and context to the discussion about the practical relevance of social presence. The implications of these findings as well as limitations of this study are discussed.

## Introduction

Given the increasing importance of online-based teaching and learning methods, mediated communication has become near-ubiquitous in many learning experiences. Where physically co-located learning offers a rich social context and many ways to interact with peers, online and distance learning relies on technology for mediation (Hillmann et al., 1994; Weidlich et al., 2018). These conditions make it particularly important - and challenging - to consider psychosocial aspects in online distance learning (Boling et al., 2012; Kreijns et al., 2003). As a result, concepts and theories related to the social realm are popular and heavily researched in the online and distance learning literature.

One particularly prominent concept is social presence, which refers to the degree of salience of the other person in the communication (Short et al., 1976). However, it is also a controversial concept, with conflicting takes regarding the definition and measurement of social presence (Lowenthal & Snelson, 2017; Kreijns et al., 2021), effects of social presence (Wise et al., 2004; Weidlich et al., 2018), as well as predictors and mechanisms of the emergence of social presence (Weidlich et al., 2019). Though researchers commonly refer to *social presence theory*, at this point, it does not seem that the understanding of the concept has reached this kind of maturity yet. Aside from the *what* (definition and measurement), the *how* (predictors and mechanisms), and the *why* (effects on interpersonal relationships and learning experiences), a mature theory should also be able to explain the *who*, *where*, and *when*, thus allowing for predictions with a certain degree of nuance (Whetten, 1989).

This study aimed to contribute to the development of a theory of social presence by introducing two additional variables, *social presence divergence* and *interaction integration*. These contextual variables add nuance by explaining when and how effects of social presence may be particularly salient or, conversely, dampened in online distance learning. To this end, we first reviewed the literature to highlight the need to consider additional variables in our theorizing about social presence and attempted to trace back their antecedents in the literature. Then we present research questions and outline the study set out to answer them. The results of this study are reported and discussed in light of the research questions. We conclude with the limitations of the study and suggest prospects for future research.

## Social Presence

In 2017, Öztok & Kehrwald (2017) wondered if it may be time to “kill” social presence, that is, to rid ourselves of an ill-defined notion and find alternative concepts and theories to help us understand mediated learning experiences. This is not the first call to action. In 2003, Biocca et al. noted the need for a more robust theory of social presence, reporting inadequacies in definition, measurement, and explanatory power of the concept. In the meantime, there has been substantial progress in many areas that were previously found lacking. For example, issues of conceptualizing and measuring social presence have been addressed (Weidlich et al., 2018; Weidlich et al., 2021). Also, social presence has been embedded in a nomological net of variables that, together, explain processes that lead to social presence as well as some of the consequences associated with social presence (Kreijns et al., 2013; Weidlich & Bastiaens, 2017). This model accommodates the *sociability* of the learning environment (S), social *interaction* among students (I), perceptions of social *presence* among students (P), contributing to a sound social *space* (S), hence the name *SIPS model*. The model has since found support in a replication of its overall structure (Göksun, 2020), experimental confirmation of a central hypothesis (Weidlich et al., 2019), and has been extended to account for a broader set of factors (Weidlich & Bastiaens, 2018).

Aside from the more recent focus on explaining the emergence of social presence, an extensive literature on effects associated with social presence has accumulated in the past decades. Here, the two variables most commonly investigated are satisfaction and perceived learning. In their meta-analysis, Richardson et al., (2017) identify large correlations of 0.56 and 0.51 between these outcome variables and social presence, respectively. It should be noted, however, that these estimates are based on primary studies that have employed a variety of different measures of social presence. While this is likely true of many educational and psychological constructs, the history of convoluted definitions of social presence suggest that these differences in measurement are not merely superficial but instead capture different constructs to varying degrees (Kreijns et al., 2021). As a result, estimated associations and effects cannot be confidently attributed to only social presence but may also be the result of implicitly measuring variables like sense of community. Indeed, Richardson et al., (2017) find the measurement scale to be a significant moderator of the association with satisfaction; however not so for perceived learning.

Another fundamental issue in our understanding of the effects of social presence lies in the conceptual setup of many empirical studies. By estimating coefficients between social presence and variables of interest, researchers fail to consider the likely context-dependent nature of these relationships. Surely the relevance of social presence for satisfying learning experience is unlikely to be uniform across pedagogical scenarios. In other words, there must be moderators of this relationship. Yet, the literature has produced almost no knowledge to this effect. Given the social-psychological nature of the construct, this neglect of explanatory contextual variables is surprising. Although we find some indications from the moderator analysis of Richardson et al., (2017), - suggesting discipline area, course length, and target audience to be relevant - at this point, it is difficult to generalize from these moderator effects. For example, our current theoretical knowledge does not suffice to explain the results that social presence appears less impactful in business contexts than in education or “other” (p.411) discipline areas. With the state of current knowledge, we have no basis for arguing whether this is a genuine disciplinary difference or possibly an artefact of the different research approaches between disciplines.

That is not to say that there are no traces of discussions on the contextual nature of purported social presence effects. For example, Zhan & Mei (2013) conceded that not all learning tasks or courses may necessitate a high degree of social presence. Depending on context, other scholars state that “[s]ometimes a low level of social presence will do the work” (Cui et al., 2013 p.676). Similarly, there have been mentions of possible threshold effects, where only once a certain degree of social presence is reached, there may be beneficial effects associated with it (Wise et al., 2004; Weinel et al., 2011). Conversely, it is also possible that crossing a hypothesized threshold may yield diminishing returns or no additional benefits at all. Finally, we could also conceive of too much social presence (Biocca et al., 2003), in which case it may be associated with negative effects (Chen, 2014); for example when interpersonal perceptions get in the way of productive learning processes. This could occur in, for example, relatively short courses in professional settings where a rich interpersonal experience serves little purpose and is considered a distraction or in learning contexts in which a certain degree of anonymity is preferred. Thus, moving away from a simple more-is-better view of social presence to one that accounts for contextual factors is a promising, yet largely unexplored perspective (Mykota, 2018). The following sections will introduce two such factors with the aim of providing generalizable and measurable additions to our theorizing into effects of social presence: social presence divergence and interaction integration.

## Social Presence Divergence

The first addition rests upon the insight that social presence effects are likely not monolithic, but instead dependent on what students would have liked to experience. Social presence divergence, thus, refers to the notion that we need not only account for the degree of social presence that is achieved, but we also need to account for the degree to which it is desirable for the students in their specific learning situation. It seems likely that preference for any level of social presence is determined by a range of different factors, many of which may differ from what is typically investigated as predictors of social presence (e.g. Cui et al., 2013; Weidlich & Bastiaens, 2018). Factors influencing preference for social presence could be, for example, learning context (academic versus professional), course duration (long versus short), students’ motivation (intrinsic versus extrinsic), instructional design (inquiry-based versus guided), and others. Similar to individual differences predicting perceptions of social presence (Weidlich et al., 2021), relatively stable traits could also account for what students would have preferred with respect to social presence.

A discrepancy between perceived and preferred levels of social presence would then lead to two possible types of divergence. The first type of divergence is what is usually referred to in the literature on social presence. Here, achieved levels of social presence are lower than what is preferred, for example when intrinsically motivated academic students take part in semester-long course but then, after a few weeks, feel socially isolated (Boling et al., 2012). A second type of divergence would be the opposite, the perceived levels of social presence are too high for what is preferred by students. This may be the case in relatively outcome-oriented courses, for example when business professionals in a hierarchical management structure are expected to complete an online-based training to acquire new workplace-related skills. Here, relative psychological distance may be preferred, for example to facilitate question-asking and to allow for mistakes and misunderstandings. Although, this type of divergence is expected to be less frequent, the possibility should be considered, as suggested by Chen (2014).

Finally, a third possible configuration is a social presence convergence. Here, the actual degree of social presence is in line with what is perceived to be desirable for the learning experience. In this situation of relative social presence convergence, students experience a degree of social presence that they judge as ideal with respect to, for example, the course design and the learning activities. In other words, there is no discrepancy. We suggest that this additional consideration – not only what students experienced but what they experienced in relation to what they would have liked - helps paint a more comprehensive pictures of social presence and its effects.

## Interaction Integration

As a second proposition, we suggest that if and how social presence divergence actually relates to outcome variables may itself be determined by another contextual factor, one that is associated with the instructional design of the learning experience. Because social presence is a phenomenon unique to mediated social interaction, the degree to which social presence divergence affects outcome variables should then be moderated by interaction integration.

Interaction integration captures the notion that learning tasks can differ in their reliance on student-student interaction (Moore, 1989) toward reaching a learning goal. For example, computer-supported collaborative learning (CSCL) is characterized by high interaction integration, because reaching learning goals is highly dependent on the successful interaction among groups of students. In other words, in these scenarios social interaction is a necessary but not sufficient condition for collaborative learning; learning fails without successful interaction (Johnson et al., 1985). On the other hand, while non-collaborative learning tasks in online distance education may include opportunities for social interaction, they are not integral to attaining the learning goals (see also contextual versus designed interaction, Oyarzun et al., 2018). Students may choose to abstain from participating in chats or message board discussions without this hampering their ability to successfully complete the learning task or course. Thus, interaction integration is low. In this case, while students may find a divergence slightly irritating, they may simply choose to abstain from social interaction.

However, if interaction integration is high the situation changes, as students are more saliently confronted with this divergence. At worst, it may be an obstacle in the way of attaining desired learning goals. For example, if achieved levels of social presence are too low in a semester-long course that relies on functioning group dynamics to complete a complex project, students may feel that this divergence actively impedes the groups’ ability to complete the project. Thus, even a relatively slight divergence may be exacerbated by very high interaction integration. In these scenarios, achieving social presence convergence would be critical and, thus should be prioritized, possibly above other design considerations under conditions of limited resources.

## Research questions

As the notion of considering perceived social presence in light of preferred social presence is, to our knowledge, absent from the literature, our first research question investigates the potential merits of this additional dimension of consideration:

RQ1: *What is the relevance of social presence divergence for understanding outcomes associated with social presence?*

RQ1.1: *How did students perceive social presence divergence in the course?*

RQ1.2: *How is social presence divergence associated with outcome variables?*

RQ1.3: *How can social presence divergence explain outcome variables above and beyond perceived social presence?*

Once we understand the relevance of social presence divergence, we can continue to add nuance by investigating the contextual conditions that may be consequential for social presence divergence (or convergence). Interaction integration is such a contextual condition that is implied by the social presence literature but has not been explicitly investigated. For this reason, our second research question is:

RQ2: *What is the role of interaction integration for understanding the relevance of social presence divergence with respect to outcome variables?*

RQ2.1: *How did students perceive interaction integration in the course?*

RQ2.2: *What is the role of interaction integration in moderating the relationship between social presence divergence and outcome variables?*

## Method

### Procedure

Survey data was collected in a compulsory course on instructional design within in a teacher education program at a Turkish higher education institution. Pre-service teachers take this course to learn about information technologies in education, theories of educational technology, new developments in teaching with technology, and evaluation of technology-enhanced learning experiences. As such, the course is designed to cover an array of theoretical aspects but, at the same time, to be applicable to classroom practice. As data collection took place during the spring semester of 2021, the course was taught via emergency remote teaching, due to the Covid-19 pandemic. To ensure interaction and quality learning experiences, several communication channels and learning activities were in place to provide a rich and varied learning experience. Students and teachers communicated via synchronous as well as asynchronous channels, as there were regular online meetings and message board discussions that took place via institutions’ Learning Management System. Students were informed about the research project during a teaching module concerned with gamification and flipped learning. They were asked for participation in data collection at the end of a 2-hour synchronous session via Zoom, in which they were presented with learning content, the opportunity to assess their knowledge through a quiz, and a learning activity in breakout rooms, where they were tasked with analyzing and synthesizing of learning content. A total of N = 305 students participated. Of these, 213 (69.8%) indicated being women, with the remaining indicating being men. No further demographic information was collected.

### Measurement

To investigate our research questions, we measure five constructs, two of which are the outcome variables, (1) satisfaction with the learning experience and (2) perceived learning. In terms of predictors, we measure (3) the degree to which social presence was experienced during the learning activity, (4) the degree of social presence students *would have* liked, and (5) interaction integration. For satisfaction, perceived learning, and perceived social presence, we use verbatim established scales (Table 1). All measures used a Likert scale with five rating scale steps (1 = disagree … 5 = agree). For preferred social presence, we slightly adapt the social presence scale, to account for experiences that students would have liked instead of what they actually perceived. To our knowledge, there is no validated scale for interaction integration. For this reason, we developed a set of items that we found to be representative of the concept (see Appendix).

Scales for measuring our constructs of interest were translated into Turkish by three translators with different relevant expertise, one being an expert in educational technology whose mother tongue is Turkish but also speaks English fluently. A second translator comes from the area of measurement and evaluation, also speaking Turkish at first-language and English fluently. Finally, a linguist with very high proficiency in both Turkish and English. After translating independently, they discussed and resolved remaining discrepancies and agreed on the final item wording.

To assess the factor structure of the measures given that they were translated, Confirmatory Factor Analysis (CFA) was conducted on scales. To this end, a separate sample of 193 pre-service teachers were recruited and asked to respond to our translated scales. Using the well-established benchmarks of fit indices by Hu & Bentler (1999) as well as Schreiber (2008), we use multiple indices to gauge model fit. Here, we report on the *X*^*2*^*/df* ratio (good fit: ≤ 3), RMSEA (good fit: < 0.08), NFI (good fit: ≥ 0.95), and CFI (good fit: : ≥ 0.95). Results of CFA suggested well-fitting models for our constructs of interest with most indices clearing predefined thresholds (Table 1). While for satisfaction adjusted Chi-square was above the threshold, other indices were fully met. For this reason, we concluded good model fit for our main variables. Aside from factor structure, four of our measures showed high internal consistency (> 0.9) while interaction integration was somewhat lower but nevertheless well above what is usually considered the cutoff for Cronbach’s Alpha (> 0.7).

Social presence divergence was calculated by subtracting perceived social presence from preferred social presence. Thus, the variable has a negative value if perceived social presence is less than what students would have liked in this learning scenario. Conversely, a positive value indicates that perceived social presence was higher than what students wanted. A value of zero indicates full convergence.

**Table 1:** Summary of measures, including results of CFA

				CFA fit indices				Cronbach’s Alpha	
	# of items	Definition	Source	*X* ^*2*^ */df*	RMSEA	NFI	CFI	Pretest Sample	Final Sample
Perceived social presence	10	The psychological sensation of the other being “there” in the communication, i.e. the perception of non-mediation	Verbatim from Anon (2018)	104.11/35 = 2.97	0.04	0.93	0.95	0.95	0.94
Preferred social presence	10	Preference for a degree of social presence within this learning scenario/activity	Adapted from Anon (2018)	103.17/35 = 2.95	0.03	0.92	0.93	0.97	0.95
Interaction integration	6	Perception of the degree to which learning success is conditional on successful student-student interaction.	Newly developed	65.76/26 = 2.53	0.02	0.91	0.93	0.78	0.76
Satisfaction	4	The extent to which students are content with all aspects of the learning experience	Anon (2017)	10.25/2 = 5.12	0.01	0.99	0.99	0.93	0.92
Perceived Learning	4	The extent to which students feel they have acquired new knowledge about the class topic	Anon (2017)	2.01/2 = 1	0.04	0.99	1	0.93	0.92

### Analysis

To understand students’ perceptions of social presence divergence and interaction integration (RQ1 & RQ2), we first explored these variables on a descriptive basis by inspecting, among others, central tendencies, comparing subgroups, and producing graphs. To account for the potential existence of gender differences with regards to social presence divergence and interaction integration, statistical comparison of gender was done via a Mann-Whitney-U test as these subgroups were unequal in size, making equality of variance unlikely.

To assess the relevance of social presence divergence with respect to outcome variables, we then first calculated bivariate correlations (H2.1) between them. Although some variables were negatively skewed (i.e. desired social presence, satisfaction, perceived learning), only desired social presence exceeded the value of -1 (skewness = -1.1). Thus, all variables remained below common cutoffs; i.e., between − 1.5 and 1.5 (Tabachnick & Fidell, 2013) or -2 and 2 (Trochim & Donnelly, 2006), allowing us to provide parametric Pearson correlations.

Then, to assess added explanatory power (change of *R*^2^) from considering social presence divergence alongside perceived social presence (H2.2), a hierarchical regression was conducted. This analysis was run twice to account for both dependent variables, satisfaction and perceived learning. For these analyses, assumptions of non-collinearity and heteroskedasticity were fully met. However, for the model predicting satisfaction it appeared that normality of residuals was not present. As our sample size was by order-of-magnitude larger than what is usually considered the threshold for the central limit theorem (n = 30), we considered this issue negligible for our purposes.

To investigate the potential moderating role of interaction integration (H3), we ran a linear regression with the two independent variables social presence divergence and interaction integration, as well as their product as interaction term. Further simple slopes analyses with interaction integration as moderator yielded information about moderator levels (Mean − 1SD, Mean, Mean + 1SD) at which associations are augmented or attenuated. These analyses were also run twice to account for both dependent variables.

We report the results of all analyses that were run twice in light of a manual Bonferroni correction, with an adjusted alpha level of 0.05/2 = 0.025.

### Power analysis

To assess the sensitivity of our statistical tests to detect significant results, we conducted a sensitivity analysis using G*Power version 3 (Faul et al., 2007). As our most complex analysis amounts to what is a linear multiple regression, we chose this from the F test family, set a significance threshold of 0.05, aiming for statistical power of 0.8, with a sample size of N = 305 and three predictors. This yielded statistical power to detect an effect size of f^2^ = 0.036, which corresponded to a small effect size according to Cohen (1988). With the Bonferroni-adjusted alpha level, this analysis is powered to detect a slightly larger effect size of f^2^ = 0.042.

## Results

**How did students perceive social presence divergence in the course?**

**Fig. 1:**
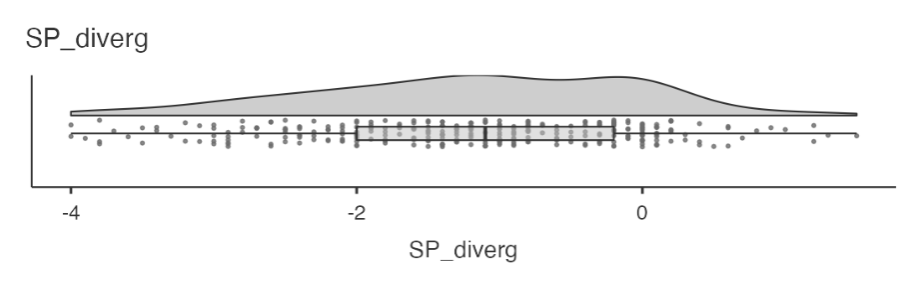
Horizontal violin plot with integrated boxplot and all data points for social presence divergence

Descriptive analysis of the data suggested that most students in this online course experienced a social presence divergence where they would have liked more social presence. This is implied by a negative mean value of − 1.19 and a median of − 1.1 (see Fig. 1). A total of n = 250 students (82%) report this type of divergence, with values ranging from − 0.1 (minor divergence) to − 4 (severe divergence). Conversely, n = 36 students (12%) reported that they experienced a higher degree of social presence than they would have preferred, implying the second type of social presence divergence (see Table 2). Here values ranged from 0.1 (minor divergence) to 1.5 (moderate divergence). Full social presence convergence was reported for n = 19 students (6%). Applying a more lenient criterium for social presence convergence that allows a slight misalignment (i.e., values between − 0.5 and 0.5,), we find that n = 87 students (29%) reported this type of rough social presence convergence.

**Table 2:** Summary of social presence divergence types

Criterium	Social presence divergence	Definition	*n*	*M*	Min / Max	*SD*	*M*_SP_ (*SD*)
exact	Divergence Type 1	< 0	250	-1.52	-4 / − 0.1	0.96	3 (0.98)
	Divergence Type 2	> 0	36	0.43	0.1 / 1.5	0.40	3.6 (0.83)
	Convergence	= 0	19	-	-	-	4.1 (0.71)
rough	Divergence Type 1	< − 0.5	207	-1.78	-4 / − 0.6	0.84	2.7 (0.83)
	Divergence Type 2	> 0.5	11	0.95	0.6 / 1.5	0.32	3.7 (0.75)
	Convergence	[-0.5, 0.5]	87	− 0.08	− 0.5 / 0.5	0.23	3.8 (0.8)

Further inspecting potential gender differences in perceptions of social presence divergence, we found that women on average reported a more severe divergence (M = -1.32) than men (M = − 0.91). This difference is statistically significant, Mann-Whitney U(213, 92) = 7629, p = .002. However, gender did not account for differences in perceived social presence, Mann-Whitney U(213, 92) = 9133, p = .347. This implies that gender difference in social presence divergence are driven mainly by preferred social presence, as reflected in a statistically significant mean difference, Mann-Whitney U(213, 92) = 7903.5, p = .007, with women preferring higher degrees of social presence (M = 4.3) than men (M = 4.02).

**How is social presence divergence associated with outcome variables?**

To probe associations of social presence divergence with affective outcome variables, we calculated bivariate correlations using Pearson’s r (see Table 3). Results showed that perceived social presence, preferred social presence, as well as social presence divergence correlated significantly positively with both satisfaction and perceived learning. With coefficients of 0.44 and 0.43, perceived social presence yields the largest association with satisfaction and perceived learning, respectively. With coefficients 0.21 and 0.14, social presence divergence shows smaller but nonetheless significant associations with these outcome variables.

**Table 3:** Zero-order correlations between measured variables

		1	2	3	4	5
Perceived SP (1)	Spearman	-				
	p - value	-				
Preferred SP (2)	Spearman	0.22	-			
	p - value	< 0.001	-			
SP divergence (3)	Spearman	0.72	− 0.52	-		
	p - value	< 0.001	< 0.001	-		
Satisfaction (4)	Spearman	0.44	0.25	0.21	-	
	p - value	< 0.001	< 0.001	< 0.001	-	
Perceived Learning (5)	Spearman	0.43	0.34	0.14	0.84	-
	p - value	< 0.001	< 0.001	0.014	< 0.001	-

**How can social presence divergence explain outcome variables above and beyond perceived social presence?**

In order to examine the predictive power of social presence divergence in explaining outcomes above and beyond perceived social presence, two hierarchical regression were calculated (one for each outcome). To avoid confusion, we do not introduce the social presence divergence variable itself into the model but instead preferred social presence. This is because social presence divergence is directly derived from perceived social presence and preferred social presence and, thus, holds no further informational value. In other words, in terms of estimation, it does not matter whether we introduce social presence divergence or preferred social presence in addition to perceived social presence in model 2 as both yield the same results.

Results showed that perceived social presence significantly explained both satisfaction as well as perceived learning, with *R*^*2*^ = 0.19 and *R*^*2*^ = 0.43, respectively (see Table 4). The addition of preferred social presence further yielded a statistically significant increase in the ability to explain satisfaction and perceived learning, to *R*^2^ = 0.22 and *R*^*2*^ = 0.25, respectively. This increase remains significant at the Bonferroni-adjusted alpha level of 0.025. Thus, social presence divergence provides a better explanation of these outcome variables than perceived social presence by itself.

**Table 4:** Hierarchical regressions to account for explanation of preferred SP on two outcome variables

	Satisfaction					Perceived Learning			
	Model 1		Model 2			Model 1		Model 2	
	*ß*	*t*	*ß*	*t*					
Perceived SP	0.42	8.5***	0.39	7.8***		0.39	0.84***	0.34	7.4***
Preferred SP			0.18	3.0***				0.28	4.9***
Model coefficients	*R* = .44 *R*^2^ = 0.19		*R* = .47 *R*^2^ = 0.22			*R* = .43 *R*^2^ = 0.19		*R* = .5 *R*^2^ = 0.25	
Model comparison	△*R*^2^ = 0.03, F(1, 302) = 8.93, *p* = .003					△*R*^2^ = 0.06, F(1, 302) = 24.7, *p* <.001			

**How did students perceive interaction integration in the course?**

**Fig. 2:**
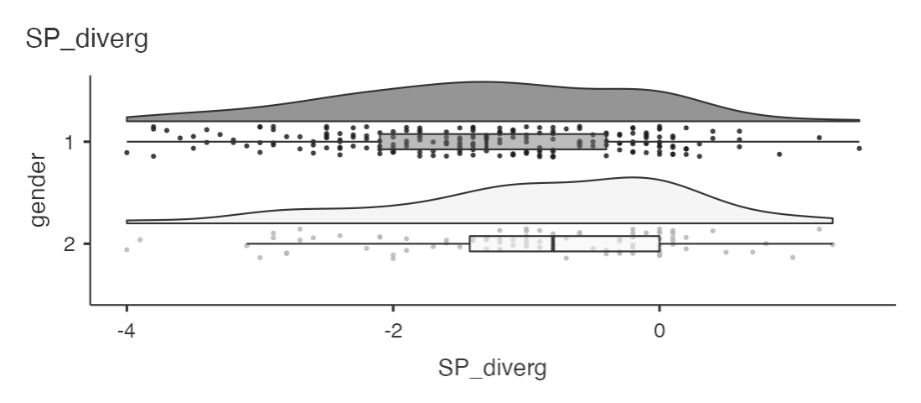
Horizontal violin plot with integrated boxplot and all data points for social presence divergence, grouped by gender. 1 = identified as women; 2 = identified as men

Turning to interaction integration, we found students reporting relatively high values of this variable, with a mean of 3.49 (*SD* = 0.75) and a median of 3.4 (see Fig. 2). This implies that, within the learning design from which these students were sampled, they perceived interaction with fellow students to be integral to achieving their learning goals. Further inspecting potential sex differences in perceptions of interaction integration, we find no mean difference between women (*M* = 3.47) and men (*M* = 3.54).

**Fig. 3:**
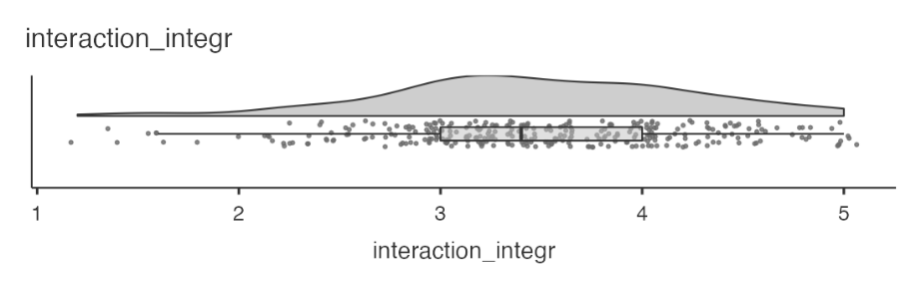
Horizontal violin plot with integrated boxplot and all data points for interaction integration

**What is the role of interaction integration in moderating the relationship between social presence divergence and affective outcome variables?**

In order to investigate the potential moderating role of interaction integration on the relationship between social presence divergence and satisfaction, we conducted a linear regression with satisfaction as criterion and social presence divergence and interaction integration as predictor variables. In addition, we enter the product of these to account for the interaction effect of interest. Due to high collinearity statistics as a result of this addition (VIF of interaction term = 17.13), we centered our predictors in order to be able to report interpretable intercepts and estimates (VIF of interaction term = 1.02). Results suggested a statistically significant interaction effect (see Table 5). This model yielded an *R*^*2*^ = 0.11. We repeated these analyses for the second outcome variable, perceived learning, finding similar results. This model yielded an *R*^*2*^ of 0.09. Both suggest a significant interaction effect of social presence divergence*interaction integration on perceived learning (see Table 5). However, these coefficients fail to remain statistically significant after adjusting for multiple comparisons, with p-values slightly exceeding 0.025.

**Table 5:** Multiple regression with interaction term

	Satisfaction				Perceived Learning			
	Estimate	SE	*t*	*p*	Estimate	SE	*T*	*p*
Intercept	4.0	0.05	79.1	< 0.001	4.0	0.05	83.7	< 0.001
SP divergence (cent.)	0.18	0.05	4.04	< 0.001	0.12	0.04	2.72	0.007
Interaction integration (cent.)	0.27	0.07	4.02	< 0.001	0.28	0.06	4.4	< 0.001
SP divergence*Interaction integration	0.11	0.05	2.13	0.034	0.10	0.05	1.99	0.047

Further simple slopes analysis showed that the relationship is non-significant at -1SD levels of interaction integration but as estimates become larger, reaches statistical significance around the mean and increases further at + 1SD (Table 6). Similar to satisfaction, simple slopes of the model predicting perceived learning showed that the relationship is non-significant at -1SD levels of interaction integration but increases at higher levels of interaction integration, reaching statistical significance around the mean and increasing further at + 1SD (Table 6). These patterns hold under the Bonferroni-adjustment of the significance level.

**Table 6:** Simple slopes analysis with three moderator levels

		Satisfaction							Perceived Learning					
Moderator levels		Est.	SE	Lower 95%CI	Upper 95%CI	*t*	*p*		Est.	SE	Lower	Upper	*t*	*p*
Mean – 1SD		0.10	0.06	− 0.01	0.21	1.79	0.075		0.04	0.05	− 0.06	0.15	0.81	0.42
Mean		0.18	0.05	0.09	0.27	4.04	< 0.001		0.12	0.04	0.03	0.20	2.72	0.007
Mean + 1SD		0.27	0.06	0.14	0.39	4.22	< 0.001		0.19	0.06	0.07	0.31	3.18	0.002

**Fig. 4:**
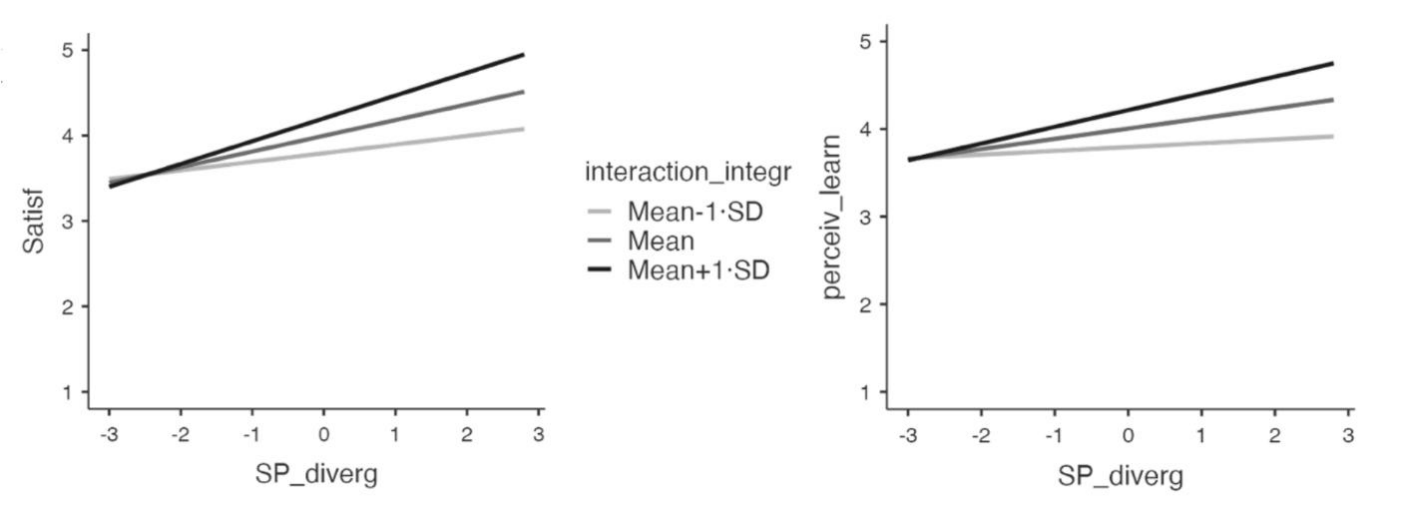
Simple slopes graph, showing the association of social presence divergence with outcome variables at different levels of interaction integration

Together, these results suggested that the relevance of social presence divergence for satisfying and subjectively effective learning experiences increases with higher levels of interaction integration. In other words, the more students felt that achieving their learning goals is conditional on successful interaction and communication with peers, the more it mattered whether social presence was aligned with what students preferred.

## Discussion

Deepening our understanding of the role of social presence in online distance learning can be achieved by increasing the nuance with which we make predictions about the outcome variables associated with social presence. In other words, a better understanding of when we can expect benefits of social presence and when we should not. To this end, we extended our theorizing via two contextual variables, social presence divergence and interaction integration.

### Discussion of the first research question

Regarding the question *What is the relevance of social presence divergence for understanding outcomes associated with social presence?*, we showed that students indeed vary with respect to how a learning scenario converged with their preference in terms of social presence. In our sample of pre-service teachers, we observed the three previously hypothesized categories, (1) a divergence where perceived social presence was insufficient, (2) a divergence with an excess of perceived social presence, and (3) a convergence of perception with preference. Notably, only a small group of students perceived a perfect convergence (n = 19). However, if we define social presence convergence more leniently by allowing slight deviations, we find that a substantial number of students – about 30% – perceived a rough social presence match (n = 87). As this interval [-0.5, 0.5] is similar to the size of one standard deviation of perceived social presence (SD = 0.98) and preferred social presence (SD = 0.8), we judged this to be an appropriate margin to conceptualize social presence convergence in this sample.

As expected, we found a majority of students reporting on the first type of divergence, which aligns with the common view espoused in much of social presence research, the *more-is-better* view, as implied by the broader social presence literature and synthesized in Richardson et al., (2017). However, with 20% of students in our sample – or 30%, depending on the category cutoffs – not belonging to this category, we provide evidence that this view is not exhaustive and a rather substantial share of students has not been explicitly considered in the literature on social presence. Importantly, for these students, a more pronounced experience of social presence is not desired and, thus, would likely not be beneficial. Notably, there was a statistically significant gender difference such that women reported a more pronounced social presence divergence on average. This finding is interesting with respect to earlier research finding that women perceived a higher degree of social presence in online learning (Richardson & Swan, 2003; Thayalan et al., 2012), while other studies failed to find such differences (Kim et al., 2011). However, as these studies work with different definitions and measures of social presence (Kreijns et al., 2021; Weidlich et al., 2018), comparisons should be drawn with care. Nevertheless, with our results showing no difference in perceived social presence but statistically significant differences in terms of preferred social presence – which accounted for the differences in social presence divergence –, we suggest that these potentially relevant differences warrant further investigation. More generally, these findings may contribute to the currently sparse literature on the relevance of individual differences in perceptions of social presence (Weidlich et al., 2021).

To gage the value of considering preferred social presence alongside perceived social presence, we reported the results of bivariate correlations as well as hierarchical regressions for predicting affective outcomes. In line with typical finding of high positive correlations between social presence and affective outcome variables (Richardson et al., 2017), we were able to replicate this association in our sample. However, this replication is not trivial due to the previously noted inconsistencies of conceptualizing social presence. Of note, Weidlich & Bastiaens (2017) found no such associations while operating under a narrow definition of social presence as it is used here. Going beyond perceptions of social presence, preference for social presence also showed positive associations with these outcomes, although more modest in size, implying that students who preferred higher degrees of social presence in this learning scenario also rated it to be more satisfying and effective. One interpretation of this may be that high expectations in the psychosocial domain may contribute to beneficial perceptions of the learning scenario, or vice versa. To sort out the causality of this association, this novel finding may be the basis for further investigations. Social presence divergence was also positively associated with affective outcome variables but these relationships were markedly smaller. Thus, the extent to which perceived social presence converges with students’ preferences, although not negligible, appears to be less relevant in terms of affective outcome variables than the mere perceptions of social presence.

With respect to assessing the relative value of considering students’ preference above and beyond mere social presence perceptions, our analyses suggested that this indeed allows for a better prediction of satisfaction and perceived learning, as demonstrated in significantly larger explained variance through the addition of this variable. To our knowledge, the relevance of considering preferences alongside perceptions has not been considered thus far in social presence research, making this a novel contribution that provides the foundation for further investigations. As an implication of this finding, we hold that this finding introduces nuance to the discussion about the potential benefits of social presence, suggesting a view of social presence effects that is less monolithic and more situational and context-dependent, in line with with Kehrwald (2008). Future research may be interested in identifying factors that explain why students differ in their preferences for social presence and if and how students indicating social presence may be unique. In terms of practical implications, online distance educators may consider not simply designing for a maximum of social presence in their courses, as there may be cost-benefit tradeoffs at play, but instead first assess students’ preference for social presence in a given learning scenario.

### Discussion of the second research question

Regarding the question *What is the role of interaction integration for understanding the relevance of social presence divergence with respect to outcome variables?*, we found students perceived a generally high degree of interaction integration. In other words, students in this learning scenario deemed successful interaction and communication to be integral for reaching individual learning goals. There was no significant gender difference with respect to these perceptions.

Considering interaction integration as a moderator of the association between social presence divergence and affective outcome variables, we found that the interaction term social presence divergence*interaction integration was a significant predictor of both satisfaction and perceived learning but not after adjusting for multiple comparisons. Further looking into the association at different levels of interaction integration, results suggest that the association between social presence divergence and outcomes was weaker when interaction integration is low, but was stronger for those students who deemed successful interaction to be integral to reaching their learning goals. Associations at high levels of interaction integration (+ 1SD) are highly significant, and thus robust to Bonferroni correction. This aligns well with our prediction that social presence divergence may be relatively inconsequential in learning scenarios that are less dependent on successful student-student interaction. If, on the other hand, students perceive this to be central to the learning experience, social presence divergence becomes a more important consideration and closing this discrepancy may be a priority goal for learning designers.

Although these dependencies may appear rather intuitive or straightforward, they have hardly been discussed nor investigated in the social presence literature. One example of this notion may be found in Oyarzun et al., (2018) with their distinction of contextual versus designed interaction, where the former refers to learning scenarios with opportunities for interaction, but these are not structured nor mandatory. Designed interaction, on the other hand refers to learning scenarios that are specifically designed to make use of student-student interaction, aligning this with our notion of high interaction integration. However, our understanding of interaction integration slightly diverges from this as we conceived the concept as (1) better represented by a continuum than a dichotomy and (2) a subjective experience of students and not a fixed property of the instructional design. As students in our sample essentially experienced the same learning scenario but reported varying degrees of interaction integration, we found evidence to support both propositions.

In the CSCL literature, we can also find traces of implicit assumptions that interaction integration is consequential for understanding effects of social presence. For example, when Poquet et al., (2018) explained that in MOOCs “learners are not obliged to engage in social activities or complete the assigned assessment in the course” (p. 44), they essentially stated that many MOOCs are learning scenarios with low interaction integration. But because they did not go on to assess outcome variables associated with social presence, this observation remains inconsequential aside from providing a rationale to study social presence in this context. As an example of recognizing the role of interaction integration, we can refer to the sustained research efforts in social presence and social aspects within CSCL by Anonymous and colleagues (Kreijns, 2004; Kreijns et al., 2013). The importance of social presence in CSCL is usually conveyed by referring to the integral role of social interaction for learning success in these scenarios. In other words, this line of research recognizes the importance of an adequate level of social presence (i.e. the avoidance of a severe divergence) due to the fact that interaction integration is typically high in in collaborative learning. The results of this study provide explicit support for these assumptions and add to the social presence literature by providing tools to better understand when we can expect benefits or drawbacks from social presence and how pronounced these may be with respect to satisfaction and perceived learning.

As a practical implication of these findings, we suggest that educators consider the degree of interaction integration in their learning scenarios. Although direct measurement of perceived interaction integration may be the ideal approach, it may not always be feasible in practice, especially ahead of time. For this reason, an expertise-based judgement on the interaction integration in the instructional design may be a good starting point to think about specific relevance of social presence --particularly social presence divergence– in their learning design. Based on this judgement, investments into enhancing social presence, for example by prioritizing instructional strategies to enhance social presence (Lowenthal & Dunlap, 2018; Weidlich et al., 2019) over other considerations, would be theoretically grounded and empirically supported.

## Limitations

This study has a number of limitations that we would like to report. First, the notion of social presence divergence is, to our knowledge, unexplored terrain within in the social presence literature. As such, our investigation should be seen as conversation-starter on this topic, while providing some preliminary evidence of the value of concept. That said, the intricacies of how students perceive social presence and how this relates to their preferences in online distance learning scenarios would be ideally further investigated with a mixed-methods research design (e.g. Lowenthal & Dunlap 2020). Although our quantitative results point to this as a worthy area of investigation, we suggest that these subjective student perceptions may be best understood by complementing quantitative with qualitative data, for example to uncover and explore factors that influence students’ perceptions and preferences.

Another limitation of our study is that we did not assess actual student learning but instead used affective self-report variables satisfaction and perceived learning as outcome variables. Of course, these are not synonymous with learning achievement (Sitzmann et al., 2010). On the other hand, although learning achievement lies at the heart of all learning experiences, cognitive learning gains are not the only relevant variable in online distance learning. Students associating positive subjective experiences with a learning scenario increases student persistence (Levy, 2007; Lee & Choi, 2013) and is associated with learning motivation (Joo et al., 2011), thus emphasizing the importance of these affective variables (Richardson et al., 2010). Still, future investigations should consider including cognitive learning gains as dependent variables in order to find further support for the relevance of considering social presence divergence and interaction integration in online distance learning.

Finally, interaction integration was not manipulated in this study. This implies certain limitations in the inferences we can draw from our moderator analysis. One limitation we trace back to this lack of manipulation is that the interaction terms failed to reach statistical significance after correcting for multiple comparisons. While we concede that this weakens the strength of our argument, we find support in the clear pattern emerging from simple slopes analysis consistent with our hypotheses. In future studies, it might be necessary to experimentally manipulate interaction integration in a learning design to provide a stronger contrast of experiences and increase the variance of this variable. Self-reported interaction integration could then be used as a manipulation check and as moderator variable.

## Conclusions

This study provides evidence to support an extension of our theorizing of social presence in online distance learning. By providing the means to account for social presence in light of student preferences (i.e. social presence divergence), we add nuance to the discussion and point to an area worthy of further investigation. At the same time, there has been a chasm between social presence research focused on CSCL scenarios versus research focused on “classic” online distance learning. With interaction integration, we introduce a concept that bridges this chasm and allows for conceiving of these disparate learning scenarios on a dimension that appears to be relevant for social presence research. We hope that these findings further stimulate the scholarly discussion of when, how, and to which extent social presence is relevant for delivering high-quality online distance learning experiences.

## Appendix

**Perceived social presence**.

In the learning environment used for this learning task….

… it feels as if we were a face-to-face group.

… it feels as if I deal with “real” persons and not with abstract anonymous persons.

… I can form distinct impressions of some of my fellow students.

… It feels as if all my fellow students are ‘real’ physical persons.

… I imagine that I really can ‘see’ my fellow students to be in front of me.

… my fellow students feel so ‘real’ that I almost believe that we are not virtual at all.

… all of my fellow students feel that I am a ‘real’ physical person.

… if feels as if all my fellow students and I are in the same room.

… if fells as if all my fellow students and I are in close proximity.

… I strongly feel the presence of my fellow students.

**Preferred social presence**.

In the learning environment used for this task….

… I would like it to feel as I we were a face-to-face group.

… I would like it to feel as I dealt with “real” persons and not with abstract anonymous persons.

… I would like to form distinct impressions of some of my fellow students.

… I would like to feel as if all my fellow students are ‘real’ physical persons.

… I would like to imagine that I really can ‘see’ my fellow students to be in front of me.

… I would like to it if my fellow students feel so ‘real’ that I almost believe that we are not virtual at all.

… I would like it if all of my fellow students feel that I am a ‘real’ physical person.

… I would like to feel as if all my fellow students and I are in the same room.

… I would like to feel as if all my fellow students and I are in close proximity.

… I would like to strongly feel the presence of my fellow students.

**Interaction Integration**.

When I think about the learning task(s) in this course….

… I feel that interaction with my peers was integral to achieving my learning goals.

… I feel that I needed to successfully communicate with my fellow students.

… it was designed so that interaction with others was central to the learning experience.

… I feel that if communication with my fellow students was hampered, it would impact my learning negatively.

… I feel that interaction and communication with my fellow students did not play a large role (-).

… I was important to get along with other students to succeed in this course.

**Satisfaction**.

I profited from this learning task.

This learning task fulfilled my expectations.

In general, I am satisfied with the learning task.

I wish more learning tasks were like this.

**Perceived Learning**.

I learned a lot in this learning task.

In terms of how much I learned, I would consider this learning task a success.

I feel like this learning task has prepared me for future materials and tasks.

I feel like I know much more about the learning content after having completed the learning task.
